# Seroepidemiology of Varicella Zoster Virus among children, adolescents and medical students in a referral children medical center, Tehran, Iran

**Published:** 2012-09

**Authors:** B Pourakbari, L Shahbaznezhad, N Parvaneh, S Nikkhah, S Mahmoudi, M Teymuri, AE Alyari, S Mamishi

**Affiliations:** 1 Pediatric Infectious Disease Research Center, Tehran University of Medical Sciences, Tehran, Iran; 2 Departments of Infectious Disease, Children Medical Center, Tehran University of Medical Sciences, Tehran, Iran

**Keywords:** Seroepidemiology, Varicella Zoster Virus, Children, Adolescents, Medical students

## Abstract

**Background and Objective:**

Varicella is a benign childhood infection with considerable complication in none immune adults. The aim of this study was to survey Varicella Zoster Virus (VZV) seroepidemiology in children, adolescents and medical students in Children Medical Center, Tehran, Iran.

**Material and Methods:**

In this cross sectional study, serum sample of children, adolescents 10 to 18 years old and medical students 18 to 25 years old were tested for VZV IgG with a commercial ELISA kit.

**Results:**

A total of 412 individuals who were 10 to 25 years of age participated in this study. Overall 269 individuals (65.3%) were seropositive for VZV IgG. Seroprevalence of VZV antibody increased with age of participants, from 59% in 10-11years children to 80% in 20-21 years old young adult students, except in 22-23 and 24-25 years old, whom the frequency of positive results decreased interestingly to 41.7 and 52.8%, respectively. Prevalence of positive VZV antibody between two genders was not statistically different.

**Conclusion:**

On-going monitoring of the seroepidemilogy of VZV is necessary to assess trends of infection in the community. A considerable proportion of young medical students in this study were still susceptible to VZV and consequent complications.

## INTRODUCTION

Varicella is usually a mild and self-limiting disease in healthy preschool and school aged children (1). Nevertheless severe complications like cerebellar ataxia, encephalitis, varicella pneumonia (2–4) and bacterial super infection of skin and lung (5) especially in newborns, immunocompromised patients, adults and pregnant women has been reported. Although nearly 5% of all cases of varicella are adults (6), up to 70% of mortality is reported in adolescents and adults (7).

The epidemiology of varicella differs and this might be related to differences in population density and risk of exposure, environmental and social factors, humid conditions, or a combination of all these factors (1).

Attack rate of varicella infection following a house hold contact is about 65-85% in none immune individuals (8). Immunity to VZV is complex and not yet fully understood. Antibodies which develop following the rash of varicella may play a role in immunity to varicella and persist for many years (1). With no active immunization policy, increasing number of susceptible adolescents and adults, the era with greater risk for complications is inevitable.

In this study, the seroprevalence of varicella antibodies in children, adolescents and young medical students was evaluated in a tertiary Children Medical Center in Tehran, Iran.

## MATERIAL AND METHODS

In this cross sectional study, the target population was children, adolescents and medical students aged 18 to 25 years old whom referred into medical center during 2008. Individuals who received blood or blood products for six month before study were excluded. After getting a signed informal consent about the aim of the study from students and parents of children, the questioner form including data of sex and age of the participants were recorded consequently. A 3 mls of venous blood sample obtained from individuals. Serum then separated and stored at -70° C for further testing. Commercial Enzyme Linked Immunoassay kit (Trinity Biotech, USA) used for detection of specific IgG antibodies against VZV. The assay was done in accordance with manufactures recommendations. Results were assumed positive if ratio value was more than 1.1 and assumed negative when value was less than 0.9. Equivocal samples retested. The sensitivity and specificity of kit were 99.4 %and 97%, respectively.

Data were analyzed by SPSS software version 16. Basic descriptive analyses were utilized to summarize participants > characteristics (sex and age) and VZV seropositivity. VZV seropositivity compared in both sex and 8 separate age groups (10-11, 12-13, 14-15, 16-17, 18-19, 20-21, 22-23, 24-25 years) by chi square test. P value of less than 0.05 was considered as significant.

## RESULTS

**Fig. 1:**
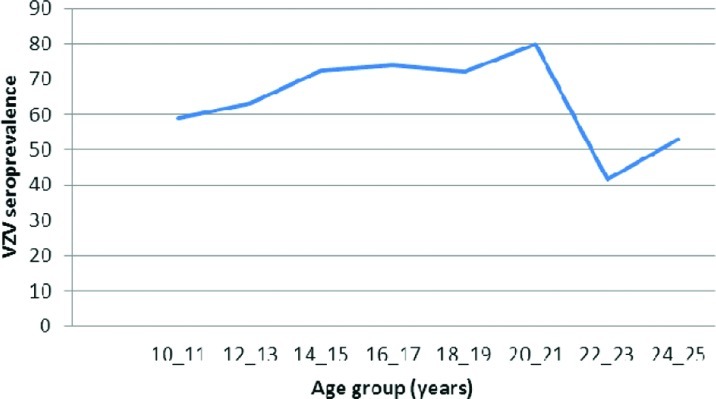
Varicella Zoster Virus seroprevalence by different age group groups

A total of 412 individuals who were 10 to 25 years of age participated in this study. Distribution of participants in separate age groups were;10-11, n = 71; 12-13, n = 76; 14-15, n = 69; 16-17, n = 54; 18-19, n = 47; 20-12, n = 35; 22-23, n = 24; 24-25, n = 36. Mean age were 164.3± years and 219 (53.2%) were male. Overall 269 individuals (65.3%) were seropositive for VZV IgG. Seroprevalence of VZV antibody was increasing with age of participants, from 59% in 10-11years children to 80% in 20-21 years old young adult students. In 22-23 and 24-25 years old frequency of positive results was decreased interestingly to 41.7 and 52.8%, respectively. Fig. 1 shows the frequency of positive VZV IgG in separate age groups. Prevalence of positive VZV antibody between two genders was not statistically different and in both groups were nearly 65% (P= 0.852).

## DISCUSSION

More than 95% of infected people with VZV develop antibody against the virus (9), which remains detectable for a long period of time (10). Finding varicella specific antibody in serum samples is an accepted approach to epidemiological studies about previous varicella infection in the community.

In our study seroprevalence rate of VZV was nearly 65%. Sharifi and Emadi (11) in 2005 reported that 83.6% of individuals 1 to 60 years in Tehran were seropositive for VZV. Ziyaeyan and colleagues from the central region of Iran, Shiraz, reported that VZV IgG was positive in 66.3% of 1-70 years old individuals (12) that is closer to our results. The rates of seropositive VZV in other countries are 87.6% in South Korea (13), 85.6% in Slovenia (8), 80.8% in Brazil (14) and 78% in Turkey that are higher than our report (15).

According to our results nearly 40% of children less than 10 years old are susceptible to VZV (seronegative) that it is similar to other published result from Iran (11). In Turkey, 92.3% of children at the age of 10 years were seropositive (15). In another adjacent country, Pakistan with nearly tropical climate, the overall prevalence of seropositive VZV is 41.5% in the age group of 6-10 years and 42.5% in the 11-15 years age group (16).

Regional and geographical difference in seroprevalence of VZV was explained previously (17, 18). In temperate climates regions where varicella vaccination has not been implemented, primary varicella infection more often occur in younger children (preschool) while in the tropical regions this infection is less frequently present in childhood (5). The incidence of varicella infection has seasonal variation and occur more often in the winter and spring (5), it may explain by the discrepancy between tropical and temperate regions.

Varicella may be considered as a nosocomial infection. Not only susceptible adult healthcare workers are at greater risk of serious complication of this disease, but also they can infect susceptible patients especially immunocompromised children and other adults. Distribution of seropositive status in medical students was notably different in our study. Although more than half of them are taking part in duties at hospitals and deal with patient care, they are still seronegative and susceptible to VZV infection. There are some studies about seropositive status of healthcare workers (HCWs). In the western region of our country, Kermanshah, 84.5% of HCW and medical students were seropositive (19). Seventy one percent of HCWs in a general tertiary hospital in Tehran were seropositive for VZV (20). In other countries the seroprevalence of varicella among medical students is greater than our reports. In Germany and Switzerland, 97% of medical students had protective levels of VZV antibodies (21, 22).

Our study shows that varicella infection occurs in late childhood. These facts may force health policy makers to pay more attention in new distributions of varicella seroepidemiology in the country.
